# Supplementary material for: Analysis of Single Nucleotide Variants in CRISPR-Cas9 Edited Zebrafish Exomes Shows No Evidence of Off-Target Inflation
Source: Front Genet. 2019 Oct 11;10:949. doi: 10.3389/fgene.2019.00949 (PMC6797590; doi:10.3389/fgene.2019.00949)
Supplement: Supplementary file 1 [file DataSheet_1.pdf]

Supplementary Material

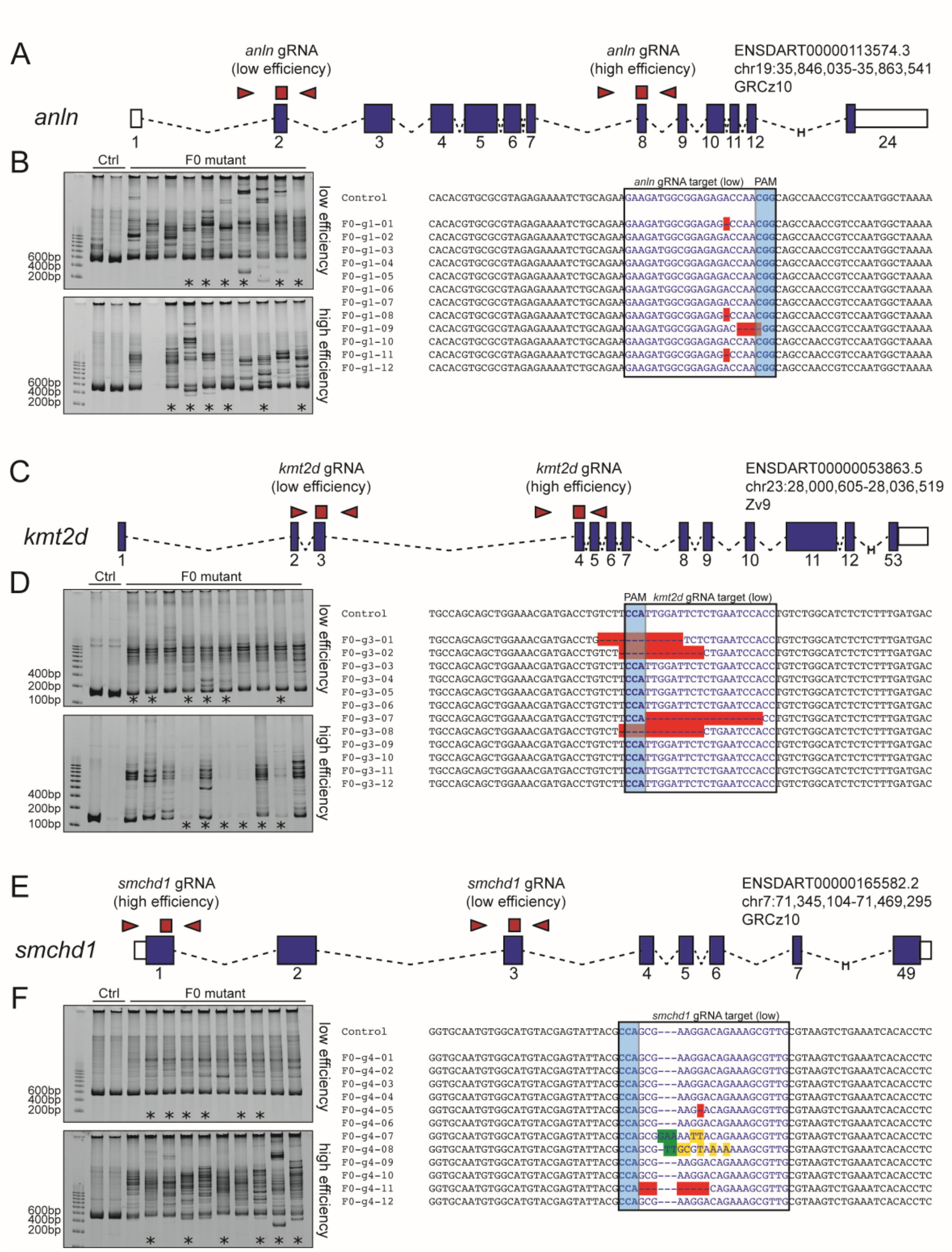

Suppl. Figure 1: Confirmation of CRISPR editing efficiencies. Efficiency data for the high efficiency guides have been published previously. (A, C, E) Schematic of the *D. rerio* locus, sgRNA targeted regions (red squares) and primers used to determine sgRNA efficiency (red triangles) for each gene of interest. (B, D, F) Heteroduplex analysis (left) and Sanger sequencing of 12 clones amplified from a single representative embryo injected with the low efficiency sgRNA plus Cas9 for each target gene (right). Efficiency was estimated by taking the average number of targeted clones across six embryos per sgRNA. * denotes samples from the heteroduplex analysis choosen for sequencing; PAM, protospacer adjacent motif.

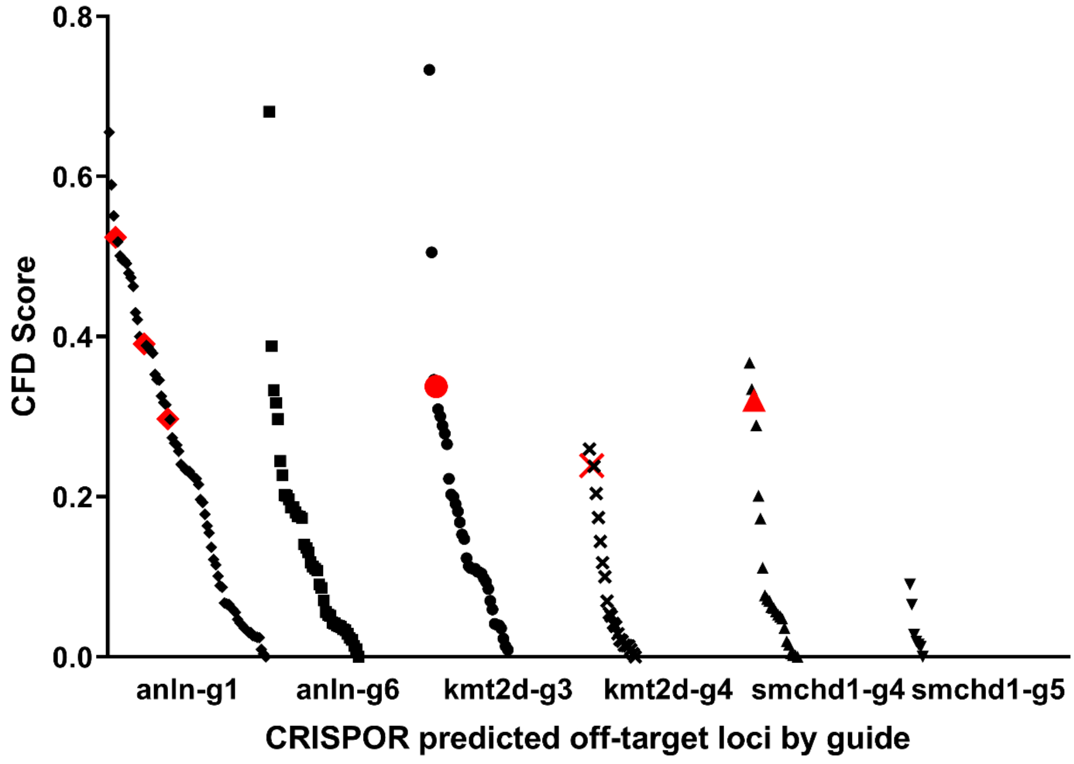

Suppl. Figure 2: CFD score distribution of off-target sequences by CRISPOR. Larger, red markers indicated loci within an exonic region and with a CFD score > 0.2.

A

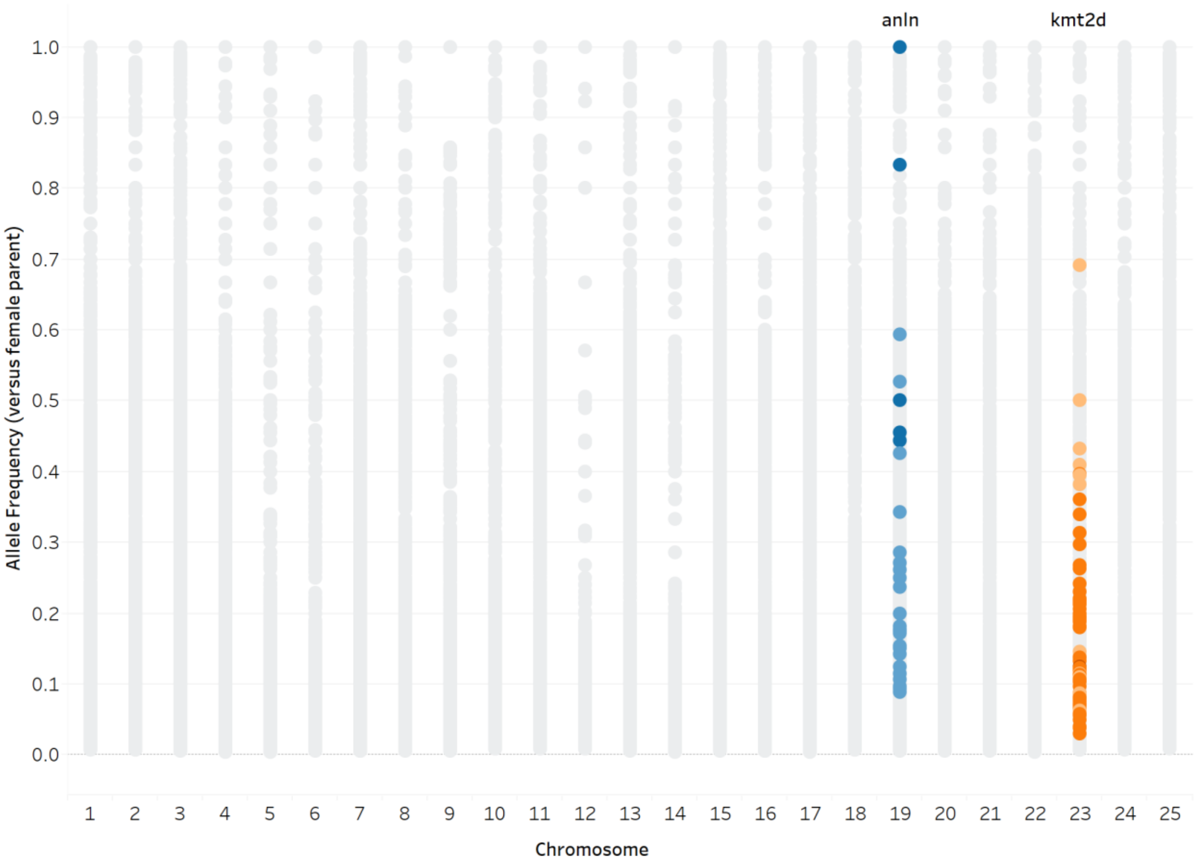

B

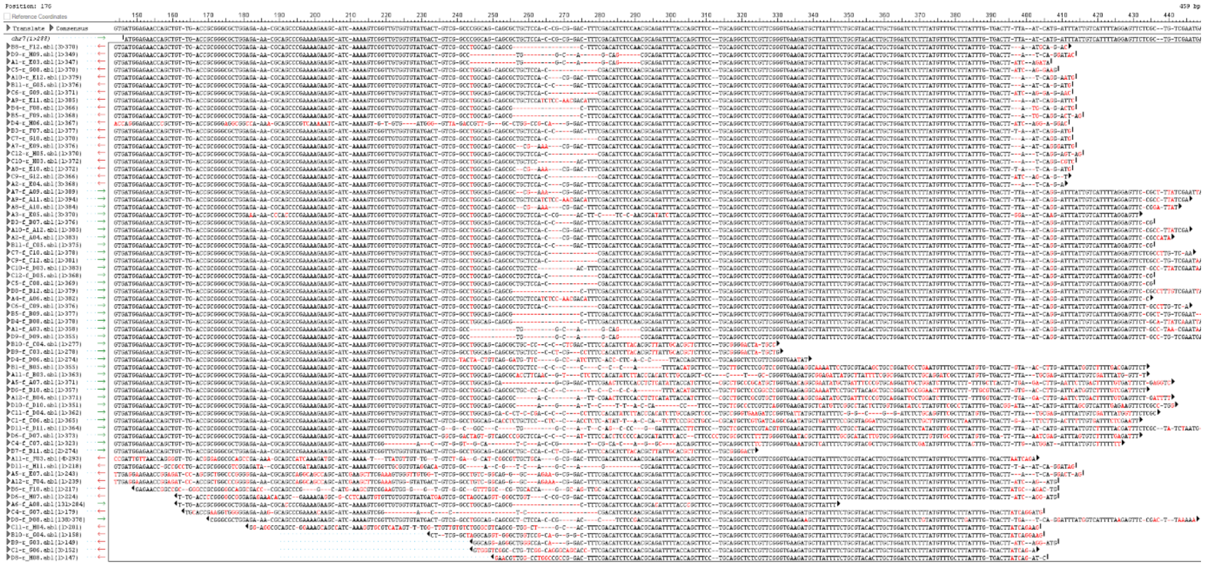

Suppl. Figure 3: On-target germline CRISPR-Cas9 editing transmitted to F1. (A) Grey circles represent all variants calls. On-target allelic series at the *anln* locus (blue) and *kmt2d* locus (orange). (B) Sequencing at the on-target *smchd1* locus in F1s originating from F0s injected with high-efficiency sgRNA plus Cas9.

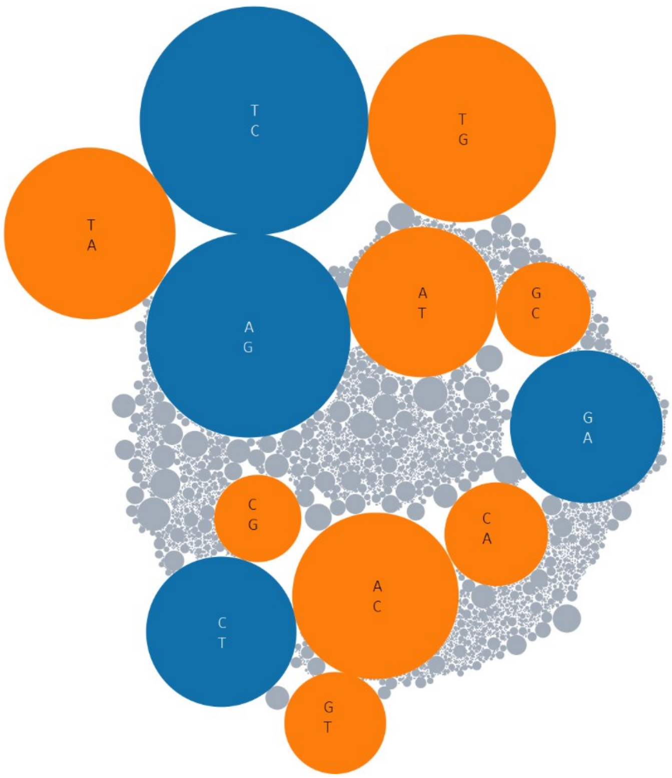

Suppl. Figure 4: Transition-Transversion ratio in F1 exomes compared to grandparental exomes. Sizes of circles represent the number of observations for each variant class: transitions (blue: 55,178 observations), transversions (orange: 60,191 observations), indels (grey: 29,319 observations). After filtering, the transition-transversion ratio is 1.09.

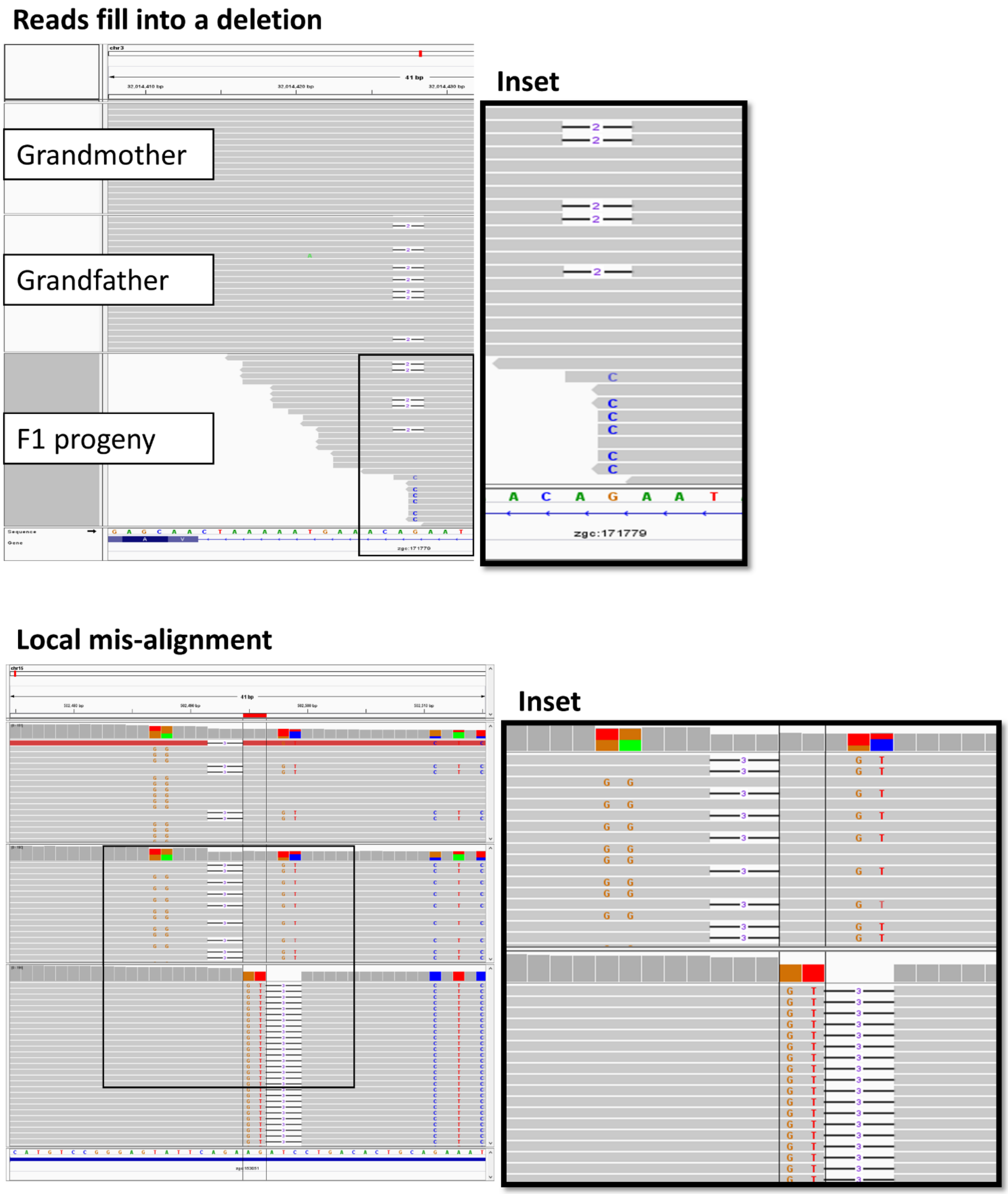

Suppl. Figure 5: Sources of systematic false positive variant calls. False positive calls in F1 progeny compared to the grandparental genotypes in IGV. Top: Reads fill in to a 2bp heterozygous deletion transmitted from the male grandparent. Bottom: A compound variant is called in the progeny due to differential placement of a 3bp homozygous deletion observed in both grandparents.

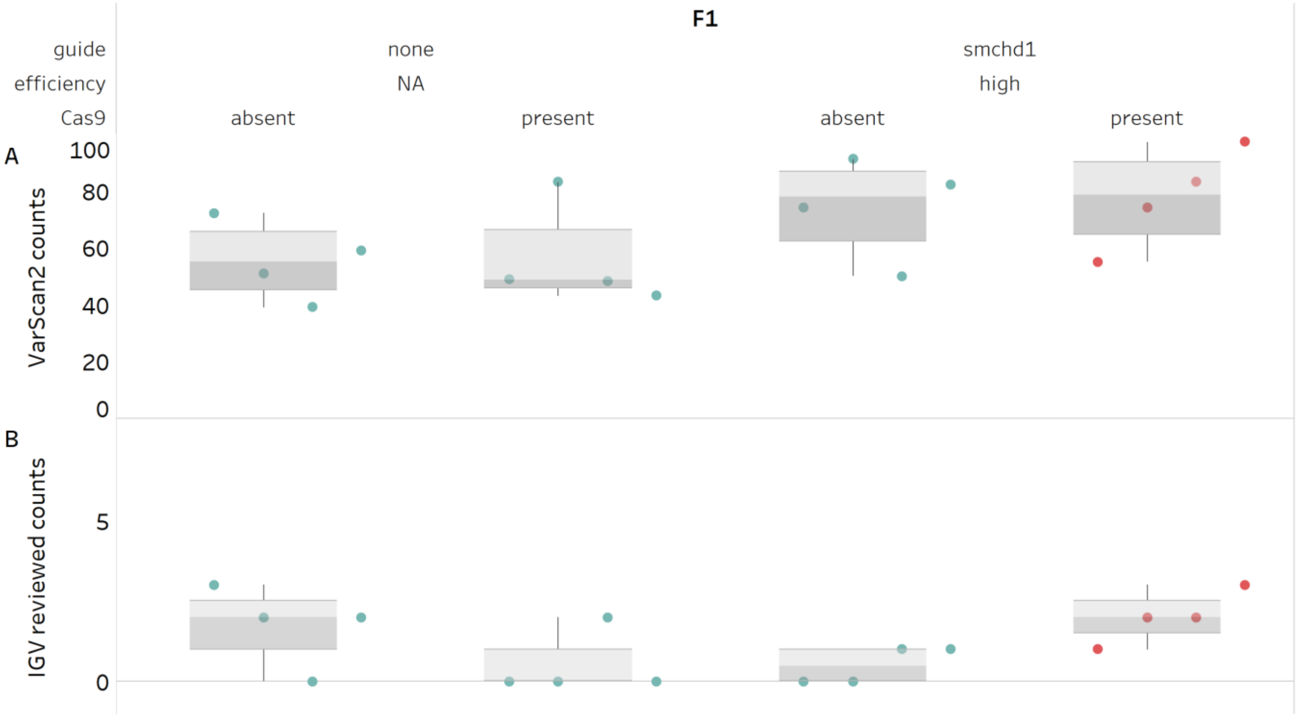

Suppl. Figure 6: Re-analysis of F1 variant count with alternative filtering strategy. A) VarScan2 variant counts passing the revised filtering strategy. B) Variants remaining after manual review for erroneous calls within an inherited deletion, local mis-realignments, or transmission consistent with low allele frequency mosaicism in the grandparents. Control individuals in green, CRISPR-Cas9 edited individuals in red.
