# Supplementary material for: Analysis of Single Nucleotide Variants in CRISPR-Cas9 Edited Zebrafish Exomes Shows No Evidence of Off-Target Inflation
Source: Front Genet. 2019 Oct 11;10:949. doi: 10.3389/fgene.2019.00949 (PMC6797590; doi:10.3389/fgene.2019.00949)
Supplement: Supplementary file 2 [file DataSheet_2.pdf]

frontiers

Supplementary Material

Suppl. Table S1: sgRNA gene targets, efficiencies, and quality score, and potential for off-targeting

Gene	sgRNA number	Efficiency (%)	Targeted genomic sequence	Genomic location	MIT quality score	# exonic predicted off-target loci by >1 caller*
**anln**	g1	Low (32)	GAAGATGGCG GAGAGACCAA **CGG**	chr19:36997110-36997132	89	5/151
	g6	High (100)	GAAGGCTTAT TATCATGCAG **TGG**	chr19:36992519-36992541	84	1/233
**kmt2d**	g3	Low (30)	GGTGGATTCA GAGAATCCAA **TGG**	chr23:28003803-28003825	79	1/99
	g4	High (100)	GGGTGAGGTG CTGATAAACG **TGG**	chr23:28007878-28007900	91	4/74
**smchd1**	g4	Low (31)	CAACGCTTTCT GTCCTTCGC**T GG**	chr7:75276576-75276598	97	4/60
	g5	High (100)	GAGATGTCGA AAGTCCGCGG **TGG**	chr7:75274461-75274483	99	0/99

*Total represents the union of the three off-target prediction algorithms: CRISPOR, CasOFFinder, and CRISPR-direct.

Suppl. Table S2: Variant counts during filtering in F1 individuals

	MuTect2			VarScan2		
**Sample**	**originally reported***	**exclude grand-parent & dbSNP**	**passing filters + AF ≥ 0.3**	**originally reported**	**exclude grand-parent & dbSNP**	**passing filters + AF ≥ 0.3**
**smchd1-S1**	**107784**	**16467**	**93**	**721082**	**4836**	**23**
**smchd1-S2**	**91320.5**	**14343**	**65**	**720522**	**5444**	**14**
**smchd1-S3**	**93910**	**14820**	**75**	**722006**	**5175**	**23**
**smchd1-S4**	**98067**	**16162**	**77**	**721454**	**4983**	**19**
**smchd1-Cas9-S5**	**99133.5**	**15695**	**70**	**722267**	**5152**	**28**
**smchd1-Cas9-S6**	**98879**	**18379**	**47**	**722224**	**5048**	**13**
**smchd1-Cas9-S7**	**90394.5**	**16366**	**79**	**722826**	**5074**	**15**
**smchd1-Cas9-S8**	**102871.5**	**15899**	**48**	**722018**	**4847**	**8**
**smchd1-g5-S9**	**111667.5**	**22892**	**82**	**721814**	**5790**	**25**
**smchd1-g5-S10**	**93602.5**	**13941**	**51**	**718963**	**5279**	**19**
**smchd1-g5-S11**	**102256.5**	**12190**	**86**	**718274**	**4909**	**23**
**smchd1-g5-S12**	**100041**	**14611**	**73**	**718720**	**5157**	**25**
**smchd1-g5-Cas9-S13**	**96334.5**	**18489**	**52**	**721700**	**5327**	**21**
**smchd1-g5-Cas9-S14**	**91999**	**18250**	**63**	**720369**	**5059**	**27**
**smchd1-g5-Cas9-S15**	**98719**	**18893**	**46**	**720855**	**5252**	**13**
**smchd1-g5-Cas9-S16**	**97144.5**	**17803**	**70**	**720601**	**5114**	**34**

*Reported count is an average of the counts called against the female grandparent and the counts called against the male grandparent.

Samples S1-S12 are control individuals, samples S13-S16 are CRISPR-Cas9 edited individuals.

Suppl. Table S3: Average count of candidate *de novo* mutations by generation and calling method

				MuTect2		VarScan2	
**sgRNA**	**Efficiency**	**Cas9**	**Condition**	F0	F1	F0	F1
**none**		absent	control	41	76	6	19
		present	control	24	60	6	16
smchd1	high	absent	control	21	72	4	23
		present	edited	26	55	4	23
	low	absent	control	27		3	
		present	edited	22		5	
anln	high	absent	control	19		3	
		present	edited	29		5	
	low	absent	control	13		2	
		present	edited	33		7	
kmt2d	high	absent	control	23		3	
		present	edited	27		3	
	low	absent	control	22		4	
		present	edited	39		7	
**Average**				**26**	**66**	**4**	**20**

Suppl. Table S4: Wilcox comparison p-values in F1

Sample	MuTect2	Varscan2
Control vs edited	0.11	0.47
Cas9 present vs absent	0.03	0.71
Guide present vs absent	0.64	0.15

Suppl. Table S5: Wilcox comparison p-values on variants called by both callers in F1

Sample	AF ≥ 0.3
Control vs edited	0.78
Cas9 present vs absent	0.57
Guide present vs absent	
High vs none	1.0

Suppl. Table S6: Variant counts in F1 individuals with and without dbSNP filter

	Original filtering strategy			Alternative filtering strategy			
**Sample**	**Original count**	**exclude grand-parent & dbSNP**	**passing filters + AF ≥ 0.3**	**Original count**	**exclude grand-parent & AF ≥ 0.3**	**non-repetitive**	**cross-noise**
**smchd1-S1**	**721082**	**4836**	**23**	**721082**	**295**	**257**	**52**
**smchd1-S2**	**720522**	**5444**	**14**	**720522**	**291**	**256**	**40**
**smchd1-S3**	**722006**	**5175**	**23**	**722006**	**311**	**293**	**73**
**smchd1-S4**	**721454**	**4983**	**19**	**721454**	**368**	**318**	**60**
**smchd1-Cas9-S5**	**722267**	**5152**	**28**	**722267**	**325**	**263**	**84**
**smchd1-Cas9-S6**	**722224**	**5048**	**13**	**722224**	**287**	**243**	**49**
**smchd1-Cas9-S7**	**722826**	**5074**	**15**	**722826**	**274**	**237**	**50**
**smchd1-Cas9-S8**	**722018**	**4847**	**8**	**722018**	**293**	**249**	**44**
**smchd1-g5-S9**	**721814**	**5790**	**25**	**721814**	**370**	**304**	**92**
**smchd1-g5-S10**	**718963**	**5279**	**19**	**718963**	**289**	**241**	**51**
**smchd1-g5-S11**	**718274**	**4909**	**23**	**718274**	**273**	**222**	**75**
**smchd1-g5-S12**	**718720**	**5157**	**25**	**718720**	**342**	**258**	**83**
**smchd1-g5-Cas9-S13**	**721700**	**5327**	**21**	**721700**	**303**	**268**	**75**
**smchd1-g5-Cas9-S14**	**720369**	**5059**	**27**	**720369**	**363**	**274**	**84**
**smchd1-g5-Cas9-S15**	**720855**	**5252**	**13**	**720855**	**278**	**233**	**56**
**smchd1-g5-Cas9-S16**	**720601**	**5114**	**34**	**720601**	**419**	**327**	**98**

Samples S1-S12 are control individuals, samples S13-S16 are CRISPR-Cas9 edited individuals.

Suppl. Table S7: Variants observed in multiple F1 individuals

Sample	Prior to cross-noise filtering	Remove between-condition cross-noise	Mis-called in deletion	Mis-realignment	Evidence in grandparent	Variants passing review
**CONTROL unique loci**		**385**				
**smchd1-S1**	**257**	**87**				
**smchd1-S2**	**256**	**99**				
**smchd1-S3**	**293**	**96**				
**smchd1-S4**	**318**	**94**				
**smchd1-Cas9-S5**	**263**	**91**				
**smchd1-Cas9-S6**	**243**	**95**				
**smchd1-Cas9-S7**	**237**	**90**				
**smchd1-Cas9-S8**	**249**	**103**				
**smchd1-g5-S9**	**304**	**89**				
**smchd1-g5-S10**	**241**	**90**				
**smchd1-g5-S11**	**222**	**55**				
**smchd1-g5-S12**	**258**	**77**				
**EDITED unique loci**		**39**	**35**	**3**	**1**	**0**
**smchd1-g5-Cas9-S13**	**268**	**21**	**20**	**1**	**0**	**0**
**smchd1-g5-Cas9-S14**	**274**	**20**	**17**	**2**	**1**	**0**
**smchd1-g5-Cas9-S15**	**233**	**21**	**18**	**3**	**0**	**0**
**smchd1-g5-Cas9-S16**	**327**	**23**	**19**	**3**	**1**	**0**
**TOTAL unique loci**	**879**	**424**				

Control samples S1-S12 and edited samples S13-S16. Variants observed more than once, but in individuals exposed to different guide and Cas9 conditions, are enumerated in as between-guide cross-noise. Variants observed more than once among individuals exposed to the same guide and Cas9 conditions are reviewed in IGV and if there is no evidence of calling error or reads in the grandparents, they are enumerated in the table as variants passing review.

Suppl. Table S8: Variant counts during filtering in F0 individuals

Sample	MuTect2			≥30x VarScan2		
	originally reported*	exclude parent & dbSNP	passing filters + AF ≥ 0.3	originally reported	exclude parent & dbSNP	passing filters + AF ≥ 0.3
uninjected-3	98730	18231	19	714178	4281	3
uninjected-5	153632.5	38063	63	724678	6727	9
Cas9-4	133284	25688	25	719479	4405	8
Cas9-5	133735	25761	24	713112	4663	5
anln-g1-1	117437.5	16151	12	706727	4899	1
anln-g1-3	140097	28430	15	720890	5970	4
anln-g1-Cas9-10	125951.5	19246	26	706488	4593	1
anln-g1-Cas9-4	109913	21248	23	712796	4419	4
anln-g1-Cas9-5	125363.5	27518	23	717865	5235	7
anln-g1-Cas9-6	152948.5	43391	84	726997	7228	16
anln-g1-Cas9-7	123259.5	19800	25	711674	4780	10
anln-g1-Cas9-9	113957.5	16139	19	704081	4497	6
anln-g6-3	122569.5	26063	17	712519	4386	1
anln-g6-4	131988.5	22096	21	710221	4236	6
anln-g6-Cas9-10	139893.5	30275	21	718040	5273	6
anln-g6-Cas9-3	116501	26021	36	713070	4956	6
anln-g6-Cas9-4	123799.5	26379	48	717711	5671	4
anln-g6-Cas9-5	132917.5	25629	28	715359	5025	4
anln-g6-Cas9-6	137644	29799	19	715692	4722	4
anln-g6-Cas9-8	120272.5	24997	25	719710	4659	6
kmt2d-g3-4	116601.5	20554	22	722869	5663	5
kmt2d-g3-5	139178	28685	23	714383	5739	3
kmt2d-g3-Cas9-1	118623	24801	60	720884	4646	10
kmt2d-g3-Cas9-2	116798	20458	46	715781	5446	7
kmt2d-g3-Cas9-4	133515.5	34500	45	722333	5807	10
kmt2d-g3-Cas9-5	111948.5	25153	32	715519	4491	5
kmt2d-g3-Cas9-6	119867.5	24248	28	719554	5316	4
kmt2d-g3-Cas9-9	133272	30346	23	720748	5624	6
kmt2d-g4-1	123010.5	31250	26	716594	5504	5
kmt2d-g4-5	124993.5	26831	21	713325	5172	2
kmt2d-g4-Cas9-4	119952.5	24033	28	707322	4837	2
kmt2d-g4-Cas9-5	119101.5	22956	20	705218	4510	4
kmt2d-g4-Cas9-6	122240	26406	31	711825	5501	2

**kmt2d-g4-Cas9-7**	**119373**	**33534**	**31**	**717861**	**5671**	**3**
**kmt2d-g4-Cas9-8**	**137530**	**33943**	**23**	**722359**	**5524**	**5**
**kmt2d-g4-Cas9-9**	**134129**	**37854**	**32**	**721268**	**5812**	**7**
**smchd1-g4-1**	**128430.5**	**26487**	**32**	**721553**	**5109**	**6**
**smchd1-g4-2**	**122734.5**	**32737**	**22**	**714473**	**5452**	**1**
**smchd1-g4-Cas9-2**	**112519.5**	**21120**	**18**	**712893**	**4548**	**1**
**smchd1-g4-Cas9-3**	**133350.5**	**28182**	**31**	**715977**	**4646**	**6**
**smchd1-g4-Cas9-4**	**129172.5**	**26522**	**17**	**709164**	**4852**	**7**
**smchd1-g4-Cas9-5**	**120364**	**26252**	**24**	**716579**	**4391**	**5**
**smchd1-g4-Cas9-7**	**125670.5**	**24490**	**28**	**718073**	**4766**	**5**
**smchd1-g4-Cas9-8**	**124193.5**	**26396**	**17**	**713050**	**4667**	**7**
**smchd1-g5-2**	**139507**	**34868**	**16**	**719051**	**5491**	**4**
**smchd1-g5-3**	**133208.5**	**32225**	**27**	**721170**	**5669**	**4**
**smchd1-g5-Cas9-10**	**125004**	**27506**	**13**	**707974**	**4411**	**3**
**smchd1-g5-Cas9-2**	**128403**	**29684**	**33**	**716875**	**4833**	**5**
**smchd1-g5-Cas9-4**	**127675.5**	**27174**	**24**	**717089**	**5516**	**4**
**smchd1-g5-Cas9-6**	**126063**	**31473**	**42**	**721368**	**5204**	**11**
**smchd1-g5-Cas9-8**	**95182.5**	**15438**	**14**	**668596**	**3244**	**2**
**smchd1-g5-Cas9-9**	**107702**	**24169**	**33**	**721002**	**6121**	**2**

*Reported count is an average of the counts called against the female parent and the counts called against the male parent.

Suppl. Table S9: Wilcox comparison p-values on predicted variants in F0

	MuTect2			VarScan2		
**Sample**	**AF ≥ 0.3**	**AF ≥ 0.1**	**All calls**	**AF ≥ 0.3**	**AF ≥ 0.1**	**All calls**
**Control vs edited**	**0.04**	**0.08**	**0.89**	**0.15**	**0.48**	**0.82**
**Cas9 present vs absent**	**0.04**	**0.15**	**0.74**	**0.05**	**0.34**	**0.89**
**Guide present vs absent**						
**High vs none**	**0.90**	**0.31**	**0.58**	**0.16**	**0.26**	**0.41**
**Low vs none**	**0.69**	**0.86**	**0.87**	**0.69**	**0.49**	**0.58**

Suppl. Table S10: Wilcox comparison p-values on reviewed variants in F0 (VarScan2 only)

Sample	AF ≥ 0.3
Control vs edited	0.38
Cas9 present vs absent	0.32
Guide present vs absent	
High vs none	0.50
Low vs none	0.61

Suppl. Table S11: Wilcox comparison p-values on variants called by both callers in F0

Sample	AF ≥ 0.3	AF ≥ 0.1	No filter
**Control vs edited**	**0.78**	**0.49**	**0.97**
**Cas9 present vs absent**	**0.96**	**0.77**	**0.51**
**Guide present vs absent**			
**High vs none**	**1.0**	**1.0**	**0.25**
**Low vs none**	**0.89**	**0.86**	**0.44**

Suppl. Table S12: Expected off-target observations in region by chance, hypergeometric p-value

Guide	Sample size (# off-target sites x 200bp)	Max. observed variants in individual	p-value (under-represented) vs population max. variant rate	p-value (under-represented) vs population min. variant rate
**anln-g1**	317200	2	1.07e-17	.531
**anln-g6**	524400	1	5.63e-32	.077
**kmt2d-g3**	269200	1	4.2e-16	.363
**kmt2d-g4**	169200	1	5.5e-10	.606
**smchd1-g4**	183400	0	NA	NA
**smchd1-g5**	151400	1	.367	.657

Hypergeometric p-values calculated with the Rothstein lab hypergeometric calculator, using the capture space (74691693 bp) as the population size, and a high or low estimate for the expected population variant counts (10975 vs 608, respectively).

Suppl. Table S13: Variants at predicted off-target loci

Sample	Condition	Variant	Ref	Alt	Variant Caller	Predicted off-target	Prediction algorithm	Allele Frequency
anln-g1-3	Guide only	24:28102298*	A	C	VarScan2	24:28102339-61	Cas-OFFinder	0.05
anln-g1-Cas9-7	Edited	24:28102298*	A	C	VarScan2	24:28102339-61	Cas-OFFinder	0.05
anln-g1-3	Guide only	2:44898792**	C	T	VarScan2	2:44898891-913	MIT CRISPOR	0.06
anln-g1-Cas9-9	Edited	2:44898798**	G	T	VarScan2	2:44898891-913	MIT CRISPOR	0.06
anln-g1-Cas9-6	Edited	7:65593684**	AC	A	VarScan2	7:65593592-602	CRISPR-direct	0.1
anln-g6-Cas9-10	Edited	16:54853302-9**	Clustered event		MuTect2	16:54853401-11	CRISPR-direct	<0.05
kmt2d-g4-Cas9-7	Edited	5:62586647**	A	ACAC	VarScan2	5:62586546-56	CRISPR-direct	0.26
anln-g6-Cas9-3	Edited	16:10603230†	C	A	VarScan2	16:10603172-82	CRISPR-direct	0.06
kmt2d-g3-Cas9-1	Edited	22:20459578†	C	T	VarScan2	22:20459646-56	CRISPR-direct	0.08
kmt2d-g3-Cas9-2	Edited	22:20459578†	C	T	VarScan2	22:20459646-56	CRISPR-direct	0.08
kmt2d-g3-Cas9-4	Edited	22:20459578†	C	T	VarScan2	22:20459646-56	CRISPR-direct	0.1
smchd1-g5-3	Guide only	2:50824997-9	CTG	AAA	MuTect2	2:50824943-53	CRISPR-direct	0.05
kmt2d-g4-Cas9-5	Edited	14:2529163	A	G	VarScan2	14:2529206-28	Cas-OFFinder	0.05
smchd1-g5-Cas9-2	Edited	25:21049069-77	ACTAGCGTG	A	MuTect2	25:21048965-75	CRISPR-direct	<0.05
anln-g1-Cas9-6	Edited	14:11156689	GAGACC	A	MuTect2	14:11156666-99	MIT CRISPOR, Cas-OFFinder	<0.05

Grey band indicates a potential off-target editing event at a predicted off-target site.

*Variant at off-target site reported in both control and edited samples for this guide
**Variant observed in siblings from other conditions
† Variant at off-target site not supported by reads on both strands
